# Barriers and levers to using an app for motor function assessment by therapists in hospitals: A qualitative study of the MFM-Play app

**DOI:** 10.1371/journal.pone.0352856

**Published:** 2026-07-31

**Authors:** Dominique Vincent-Genod, Manon Verroul, Pascal Rippert, Shotaro Tachibana, Carole Vuillerot, MFM-Play Study Group

**Affiliations:** 1 Hospices Civils de Lyon, Centre Référent Maladies Rares Neuromusculaires- Hôpital Femme Mère Enfant, Lyon, France; 2 Université Jean Monnet Saint-Étienne, Lyon 1, Université Savoie Mont-Blanc, Laboratoire Interuniversitaire de Biologie de la Motricité, Saint-Étienne, France; 3 Hospices Civils de Lyon, Pôle Santé Publique, Service de Recherche et épidémiologie cliniques, Lyon, France; 4 Université Lyon1, Pathophysiology and Genetics of Neuron and Muscle, CNRS UMR, INSERM U1315, Faculté de Médecine Lyon Est, Lyon, France; Fondazione Policlinico Universitario Gemelli IRCCS, ITALY

## Abstract

**Background:**

The Motor Function Measure (MFM) is an important evaluation tool used to monitor the progression of most neuromuscular diseases. However, the MFM is subject to inter-rater variability, and patients must produce their best effort, which can be difficult for children. In response to these issues, we developed a digital app named MFM-Play, designed to optimise completion of the MFM in a fun manner. The objective of this study was to explore barriers and levers to the MFM-Play’s use by therapists.

**Method:**

We conducted a qualitative study with rehabilitation professionals who had tested the app in a previous reliability study. Individual interviews based on the Technology Acceptance Model were followed by a focus group based on the interview results. Thematic analysis was performed on transcripts.

**Results:**

Seven therapists participated in the interviews and seven others in the focus group. The results revealed the multi-dimensional features related to adopting the MFM-Play app. Barriers related to ease of use, training and organisational constraints were identified, and were partly due to the additional mental burden of learning to use the technology. However, therapists who were at ease with the digital tool found it useful for their practice. The app also modified patient-therapist dynamics, fostering greater patient autonomy, but sometimes disrupted therapists’ habits, by calling into question assessment methods.

**Conclusion:**

The MFM-Play app offers considerable practical and efficiency benefits; however, its successful adoption depends on overcoming technical, organisational, and user-specific barriers. This involves providing support for the change in the therapist’s stance within the triadic patient-therapist-tablet relationship.

## Background

Scales measuring motor function are crucial to objectively document changes in functional ability in people with neuromuscular diseases [1–4]. They are widely used in clinical practice to monitor disease progression and adapt rehabilitation interventions. In clinical trials, functional scales are not only used to track progression but also to quantify treatment efficacy, stratify patients, and support regulatory approval, which explains their increasing importance with the development of genetic and pharmaceutical treatments for diseases such as Duchenne Muscular Dystrophy (DMD) and Spinal Muscular Atrophy (SMA) [5,6].

Several other functional outcome measures exist in the neuromuscular field, including the North Star Ambulatory Assessment, the Hammersmith scales, and functional walking tests such as the 6-Minute Walk Test [7–12]. Among the available measures, the Motor Function Measure (MFM) stands out as a validated tool applicable to most neuromuscular diseases [13,14]. Its major strengths lie in its robust psychometric properties, high sensitivity to change, and three-dimensional structure, which allows for a detailed assessment of the different components of motor function [15]. It is used internationally in clinical settings and as an outcome measure in numerous therapeutic trials, including as the primary endpoint in the SUNFISH trial in SMA (ClinicalTrials number NCT02908685) and the TAMDMD trial in DMD (NCT03354039). The MFM exists in two versions. The MFM-32 is composed of 32 items and is validated for use in people with neuromuscular diseases aged 6–60 years [13], and the MFM-20 is composed of 20 items from the MFM-32 and is validated for use in children with neuromuscular diseases aged from 2 to 7 years [14]. The average assessment time is 26 minutes for the MFM-20 and 36 minutes for the MFM-32 [13,14].

Rehabilitation professionals must be trained to use the MFM to ensure good inter-rater reproducibility. However, despite standardised training, inter-rater variability in rating persists, largely because of the intrinsic qualities of the evaluator and their experience [16]. The test requires the patient to produce their best effort; therefore, they must be motivated to participate actively. This can be challenging, particularly for children [17] and increases the therapist’s cognitive load during administration. Another issue is that frequent reference to the manual is required to see the exact tasks to be performed and the scoring method; however, clinicians sometimes believe that they know the test and develop their own habits, a phenomenon known as drift [16,18,19].

To reduce inter-rater variability, increase patient motivation, facilitate scoring without drift, and reduce therapists’ cognitive load during administration, we developed a digital app, MFM-Play, to guide completion of the MFM (both the MFM-20 and MFM-32) on a tablet. The app provides interactive animations and age-appropriate content across four modules (three for children and one for adults) (Appendix 1 in S1 Appendix). A split-screen interface shared between the rehabilitation professional and the patient shows each item as a picture, enhancing the patient’s understanding and engagement. Standardisation is reinforced by providing clear visual information and immediate access to the scoring manual, increasing the rehabilitation professional’s adherence to the assessment guidelines. This digital approach allows for a smoother administration, without pauses, making the experience more motivating and fun for young children, and more participatory and enjoyable for adolescents and adults. Although motivation is generally not an issue for older participants, the aim is to improve their evaluation experience, which could be more passive with the original MFM.

Although the use of digital and technological tools is increasing in Physical Medicine and Rehabilitation [20,21], their uptake by rehabilitation professionals remains challenging [22]. Implementing such tools successfully requires developing new technical skills and modifying organisational and cultural processes. Key facilitators include ease of use, compatibility with existing practices, and reduced barriers, including financial [23]. Although the clinical validation of an app is essential, it is not sufficient to guarantee its integration into clinical practice. To facilitate its adoption, it is necessary to understand the factors that influence the app’s acceptability from both ergonomic (e.g., ease of use and satisfaction) and social perspectives (e.g., alignment with professional values, the healthcare system, and cultural context). Moreover, digital tools change practices; therefore, an analysis of the changes required can facilitate their adoption [24,25].

The objective of the MFM-Play qualitative study was to explore barriers and levers to the app’s use.

## Materials and methods

### Design

We conducted a qualitative study to gather rehabilitation professionals’ feedback on their initial experiences with the MFM-Play app between November 2023 and August 2024. An inductive method was used to capture opinions, beliefs, and feelings expressed by individuals or groups about a specific topic [26].

The study comprised two phases. It began with individual semi-structured interviews based on the Technology Acceptance Model (TAM) [27], followed by a focus group based on the interview results. The TAM predicts whether an individual will adopt a digital app, focusing primarily on two perceptions: (1) “perceived ease of use”, defined as the degree to which using a technology will be free of effort, and (2) “perceived usefulness” described as the degree to which a person believes that using a particular system would enhance their performance [28]. Interviews were conducted until data saturation was reached. Data saturation occurs when participants’ responses no longer generate new ideas or when the size of the population surveyed no longer allows for further interviews. Generally, data saturation is reached after 15–20 interviews [29].

### Participants

The study involved rehabilitation professionals from ten hospitals who had tested the app as part of a study of the app’s reliability (ClinicalTrials.gov: NCT05227274). The centres involved included 9 neuromuscular reference centres and one clinical research laboratory, all in France. All centres conduct MFM assessments for both clinical practice and clinical studies. To ensure a rigorous and homogenous MFM completion throughout the reliability study, all the rehabilitation professionals involved had to have undergone formal training in the use of the measure and to participate in a refresher course at the beginning of the MFM-Play reliability study. To be eligible for this qualitative study, the rehabilitation professionals had to have completed more than three MFM evaluations using the app. Among the 25 rehabilitation professionals who had participated in the MFM-Play reliability study, 16 eligible participants were invited by email with an information letter. They were asked to participate in either the individual interviews or the focus group, coordinating within their department so that all participating departments would be represented in the focus group.

### Ethical and regulatory aspects

Ethical approval was obtained from the regional review board (*Comité de Protection des Personnes Ouest II)* as part of the broader approval for the MFM-Play study. All participants provided written consent prior to their participation in the study, including authorisation for audio recording. The article is reported according to the Consolidated Criteria for Reporting Qualitative Research (COREQ) [30].

### The MFM-Play app

The MFM-Play app was developed by DOWiNO Studio (Lyon, France) in collaboration with clinicians from the neuromuscular disease reference centre at Hospices Civils de Lyon. The app aims to standardise the completion guidelines for the 32 MFM items while offering age-appropriate, engaging scenarios. It comprises four modules tailored to different ages and maturity levels, from young children to adults. Items 18, 19, and 22 of the MFM are interactive: they are performed directly on the tablet, as previously validated [31].

**Fig 1:**
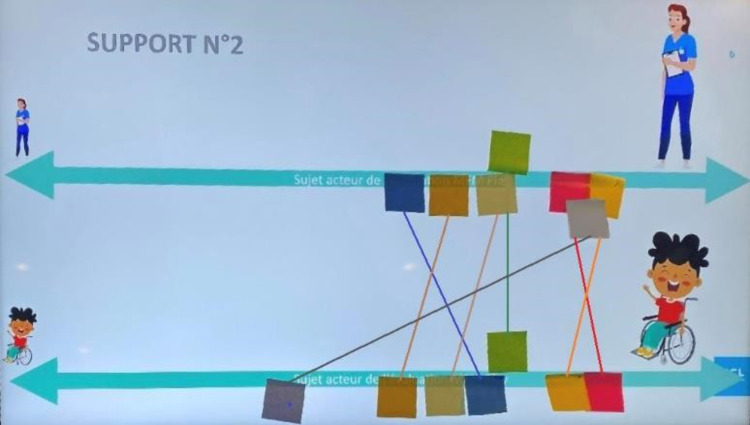
Photo of the axes used to discuss the second theme in the focus group.

**Fig 2:**
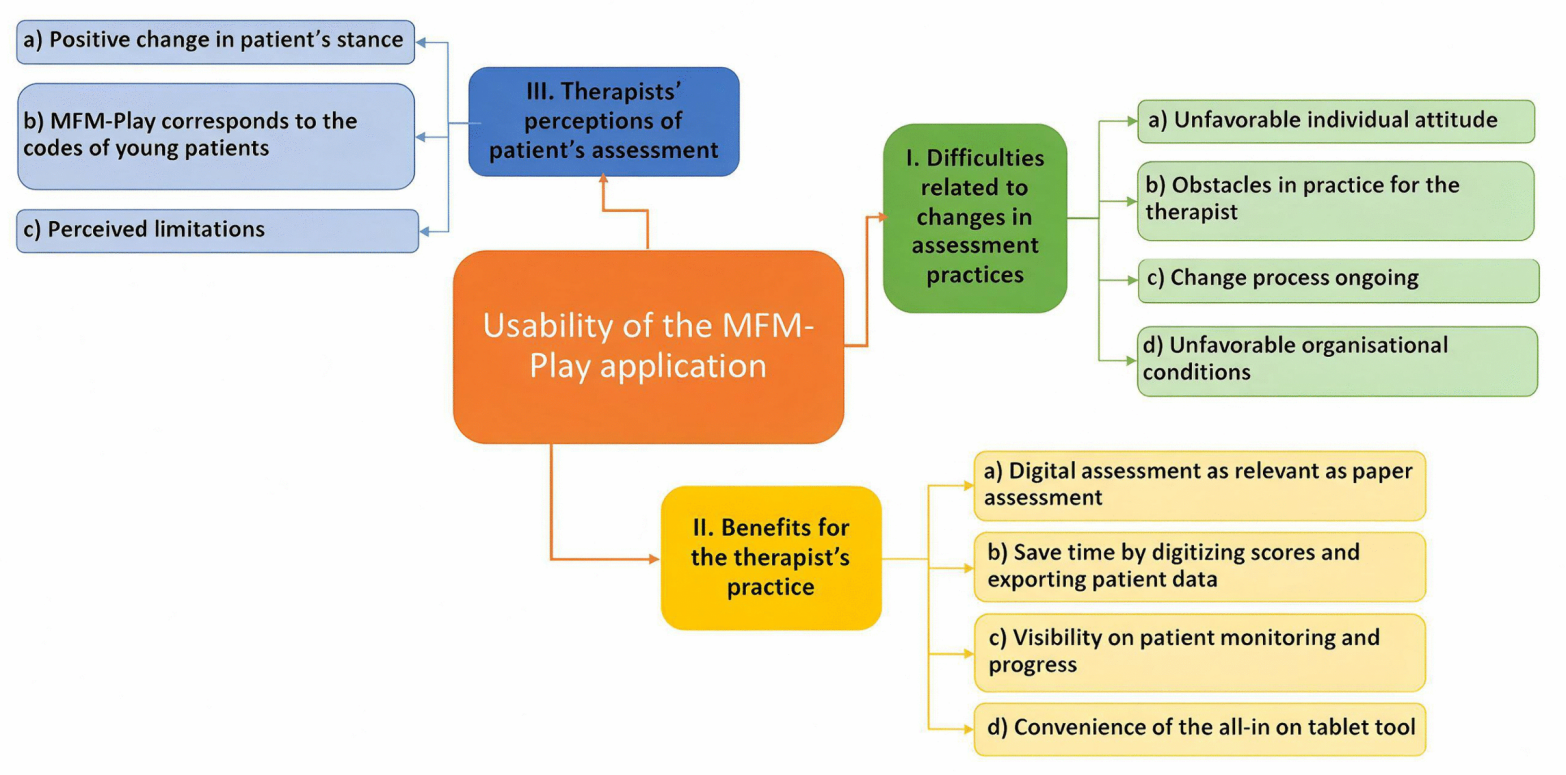
Themes identified in the individual interviews.

In the app, scenarios adapted to the patient’s age unfold in space (Appendix 1 in S1 Appendix, Fig 1). An astronaut hero and their robot companion travel from planet to planet in a spaceship. An introductory page immerses the patient in the story before displaying a map of the planets (Appendix 1 in S1 Appendix, Fig 2). On each planet, the hero experiences an adventure resolved by performing an action corresponding to an MFM item. Each item is illustrated with a picture. After completing the action, the rehabilitation professional records the score on the tablet, and an export module allows the entire evaluation to be exported in CSV format. Several story endings are possible for each item, depending on the level of success of the motor task performed. In subsequent MFM assessments, previously visited planets are identified, and rewards are awarded as collectable images, not based on performance levels (as functional capacity typically deteriorates in neuromuscular disease) but on progression through the evaluation.

### Interviews and focus group

The complementary use of individual interviews and focus groups allowed data collection at different levels: 1) Individual interviews analysed specific experiences, perceptions, and challenges in a confidential setting where participants could comfortably provide constructive feedback; 2) the focus group explored participants’ representations of the app as a group, offering diverse perspectives and highlighting common trends. Participants in focus groups build on each other’s ideas, which can bring out observations that individuals might not have expressed on their own. This method identifies common problems and highlights the main barriers and levers encountered by multiple users, offering an overall view of usage trends [32].

#### Individual interviews.

The objective of the individual interviews was to identify barriers and enablers related to the usability characteristics of the MFM-Play app, based on the experiences of rehabilitation professionals. Usability is a feature of the interaction between a user and a product. According to ISO 9241−11 (1998), usability is defined as “the extent to which a product can be used by specified users to achieve defined goals with effectiveness, efficiency, and satisfaction in a specified context of use.” Usability can be broken down into five major characteristics: efficiency to use, subjective satisfaction, ease to learn, ease to remember, and reliability (few errors), which can be considered components of effectiveness [33].

An interview guide (Appendix 1 in S1 Appendix) was developed based on the TAM model and the definition of usability in the literature [34,35], and formulated by two researchers (MV, DV). The guide structured the data collection and exchanges by defining the different themes to be addressed. It was organised into four themes: user satisfaction, effectiveness and efficiency, app adoption, and the social influence on usability.

Semi-structured individual interviews were conducted by a social psychologist (MV) who had no connections with the participants. Interviews were conducted via video conference or in person, depending on the participants’ preferences and geographical locations. The interviews lasted 31–45 minutes and were audio-recorded and transcribed verbatim to anonymise the words of each participant. The maximum number of rehabilitation professionals available within the four months preceding the focus group data analysis was included.

#### Focus group.

The focus group aimed to enable a collective reflection on the barriers identified during the individual interviews regarding the usability of the MFM-Play app and to propose levers. It also sought to capture participants’ perspectives on various factors impacting the usability of the MFM-Play app. The focus group interview guide (Appendix 1 in S1 Appendix) was developed based on these barriers: 1/ therapist barriers regarding the change in assessment practice, 2/ therapist barriers regarding the change in the relationship with the patient and 3/ difficulties or challenges perceived by the therapists concerning patients.

The focus group also aimed to gather the participants’ points of view on the levers they had already implemented or planned to implement to facilitate the use of the app.

The focus group was presented using workshop materials. To support discussion of the first theme, four verbatim interview excerpts illustrating challenges with the MFM-Play app’s usability were presented. Participants were invited to propose solutions to these issues, including strategies they may have already implemented. To introduce the second theme, we presented two horizontal axes illustrating the level of involvement of each party using the MFM-Play app: one for the rehabilitation professional and one for the patient (Fig. 1). These axes allowed participants to position the respective roles during MFM-Play completion and supported discussion about participation and responsibility—specifically, the question of “who is the actor of the evaluation.” For the third theme, the group completed two “patient profile” cards to identify factors that facilitate or hinder the use of the MFM-Play app.

The focus group lasted 106 minutes and was conducted by the social psychologist who had carried out the individual interviews (MV). Discussions during the focus group were audio-recorded and transcribed verbatim. The absence of an observer was a deliberate choice to minimise observation bias, as the presence of an observer could influence participants’ behaviour. By adhering to principles of neutrality in qualitative research [36], this approach created a more natural and spontaneous environment, allowing participants to focus entirely on their interactions with the app and with one another, without the pressure of feeling evaluated.

The seven therapists simultaneously positioned their coloured markers on a large screen showing two axes ranging from “passive participation in the MFM” to “active participation in the MFM”. Each therapist was provided with two identically coloured markers. The coloured marks were connected by lines to analyse the results.

### Analysis

Sociodemographic data and the rehabilitation professionals’ experience were summarised using descriptive statistics.

The data collected from the individual interviews and the focus group were analysed using thematic content analysis based on Bardin’s method [37]. This method involves breaking the text into units of meaning for categorisation through assigned codes. This process was repeated across all transcribed content to critically identify groupings of ideas into themes and sub-themes, excluding non-pertinent or non-representative data from participants [37]. This inductive analytical method provides a comprehensive understanding of phenomena based on raw data from individual interviews and the focus group, considering the contexts in which the data were collected [38].

The coding was inductive and focused on themes related to the acceptability of the new MFM evaluation procedure using the MFM-Play app, as reported by therapists, concerning both their own role and the patients’ role. The analysis was based on the principle of interdisciplinary triangulation to ensure the accuracy and stability of the observations [39]. Coding was conducted using Tagette software by one researcher (MV) and independently verified by another researcher (ST). To respect confidentiality, double coding of transcripts could not be carried out for the interviews, as it would not have allowed participant anonymity to be maintained. The sample was small and the participants had been identified in advance, so a second review was carried out on the coding book. Four authors (MV, ST, DV, and PR) participated in in-depth discussions to resolve disagreements on the initial coding and organise the final themes.

## Results

Among the 16 eligible individuals, 7 (3 men and 4 women) agreed to participate in the individual interviews, and 7 (2 men and 5 women) in the focus group (Table 1). Thirteen were physiotherapists and one was an occupational therapist. Two were MFM trainers.

**Table 1:** Participant characteristics of the participants in the interviews and focus groups.

Therapist ID	Participation phase	Age	Gender	Years’ experience with MFM	No. of MFM-Play completed
Int1	Interview	36	Female	6	4
Int2	Interview	41	Male	5	16
Int3	Interview	39	Male	3	3
Int4	Interview	34	Male	4	11
Int5	Interview	42	Female	8	3
Int6	Interview	48	Female	15	5
Int7	Interview	38	Female	2	4
FG1	Focus group	48	Male	9	3
FG2	Focus group	34	Male	5	18
FG3	Focus group	27	Female	2	17
FG4	Focus group	50	Female	15	5
FG5	Focus group	52	Female	10	4
FG6	Focus group	39	Female	5	6
FG7	Focus group	41	Female	13	7

The participants in the individual interviews were aged 34–48 years, and those in the focus group were aged 27–52 years, with 2–15 years’ use of the traditional MFM in both groups (Table 1).

### Thematic analysis of individual interview content

The analysis of the interviews identified three main themes: difficulties faced by rehabilitation professionals in changing their evaluation practices, advantages of this change for their practice, and professionals’ perceptions of this change for patients (Fig. 2).

### Difficulties faced by rehabilitation professionals in changing their evaluation practices

The shift in evaluation practices induced by the app was complex for several reasons. Some individuals had preconceived negative attitudes toward using the MFM-Play: *“preconceived ideas, let’s say, that it would be a hassle every time”* (Int5). The mental load associated with this change or a lack of digital proficiency also complicated the perception of the task and *“added constraints”* (Int3). Participants identified barriers in their practice, such as changes in the patient-professional dynamics induced by MFM-Play and shifts in attention and vigilance during evaluation: *“I think that at first, when we ourselves are discovering the app, we do feel a certain responsibility as therapists to do it well. So, I’d say that we do have a bit of pressure—not really stress, but we tell ourselves, ‘Okay, we need to do this properly.’ As a result, we tend to be more focused on applying it correctly rather than fully paying attention to [what the patient is doing].”* (Int2).

Participants were still in the process of learning to use the app, requiring more time, and its use, being novel and sporadic, was not yet sufficient to make it intuitive: *“it’s not every day; it’s not as frequent as the traditional MFM completion, so you have to get back into it, like, ‘where do I click again’? It’s not a reflex yet”* (Int1). This unfavourable pattern seemed to reduce the use of the MFM-Play app. Some participants questioned the reliability of the three items administered on the tablet compared to their traditional paper versions (items 18 and 19) because *“the stylus doesn’t have the same resistance as paper, and it adds a bit of height, whereas paper is flat [...] the circle too, which makes it not quite the same"* (Int1). Finally, organisational conditions were not always conducive to using the MFM-Play: some participants experienced a lack of flexibility in scheduling consultation times, *“connection issues”* (Int7) with the internet, or a lack of communication among department professionals about the app’s use.

#### The advantages of this change for their practice.

The participants highlighted several advantages for their practice. They particularly appreciated the time saved by digitising scores and exporting patient data, despite commenting about the overall increased evaluation time when using the app. The app also allowed a simplified retrospective view of the patient’s overall MFM follow-up: *“the comparison of evaluations for the same person when we’ve done several evaluations”* (Int4). Furthermore, MFM-Play consolidated nearly all the resources needed for evaluation in one place, making it an *“extremely practical [tool]”* (Int4). Finally, they considered that completion of the evaluation was as effective with the app as the traditional version: *“I perform the same evaluation [...] it feels like I’m doing the same thing”* (Int5); *“the exercises are the same with the tablet and with paper”* (Int7).

#### The professionals’ perception of this change for patients.

The participants interviewed expressed different opinions about the evaluation’s impact on patients. The use of the MFM-Play app induced a shift in stance for patients during the MFM assessment. This shift was positively described: “*patients are more active in their evaluation”* (Int1), placing them in a specific dynamic that could help them *“detach somewhat from the hospital context”* (Int3) during the evaluation. Additionally, the tool was considered to align with younger patients’ preferences by offering *“a playful approach for children through the tablet, which is often quite appealing for young people and even, at times, for adults”* (Int3). The interface allows patients to visualise the movements they need to perform, which seemed to increase their satisfaction since *“visualising the item can help some individuals understand what is expected of them”* (Int5), giving them autonomy. *“Oh well, actually, I didn’t really need to be as present to give instructions anymore, and the child engaged on their own. So yeah, there was more of this learning process of letting the child be autonomous, even though they have attention difficulties, which I think were actually well managed in the end.”* (Int2). However, certain limitations were noted by the participants, who felt that the assessment method was less well suited to certain patient profiles. Difficulties were encountered with adolescents or adults with the reading mode compared to the more appreciated audio mode: *“for older patients, where the story isn’t narrated but has to be read, we found it too long”* (Int7). Attention to the physical and psychological state of the patient during the sequence of items also made MFM completion more difficult. Thus, using the tablet could complicate the evaluation for certain profiles: *“balancing on one leg, then sitting back down, doing the story, and then standing back up for five seconds... for weaker patients or those for whom effort is very demanding”* (Int6). Additionally, the visibility of item scores was described as a potential distraction for some children.

### Thematic analysis of focus group content

Three themes emerged from the focus group discussions of the barriers and levers mentioned by the participants in the interviews: change in the therapist’s social identity, perceived lack of usability of the interface and dissatisfaction with the evaluation (Table 2).

**Table 2:** Barriers and levers perceived by the participants in the focus group.

	BARRIERS	LEVERS
**Change in the therapist’s social identity**	Change in role Poor mastery of digital tools Change in habits Organisational change	Reduces patient/caregiver asymmetry Facilitates interaction with the patient in complex situations Reorganisation of work (reminders to charge tablet, note connection codes, etc.)
**Perceived lack of usability of the interface**	Poor mastery of basic commands Lack of knowledge/ignorance of the data export functionality	Peer education
**Dissatisfaction with the evaluation**	Lack of intuitiveness in rating items Viewing of item rating by the patient Lack of link between the story and the item assessed Absence of audio mode for adolescent and adult modules Not suitable for all severity levels	Use of non-stereotypical colours Audio mode for all levels Increase the number of rewards Choice of the story universe in line with the patient’s interests Reinforce use among patients for whom MFM-Play is more suitable

#### The therapist’s social identity.

According to most participants, evaluation with the MFM-Play involves a shift in stance, raising questions about their social identity. Some acknowledged adopting a more supportive role, which reduced the initial asymmetry in the patient-therapist relationship to the point where one participant *“felt a little disengaged and replaced”* (FG-T2) by the tablet. However, as shown in Fig 1, the therapist generally remained active in the evaluation when using the MFM-Play app. The participants described a *“less top-down and less directive evaluation”* (FG-T1) process, with patients taking a *“more active role”* (FG-T4) in their evaluation.

In this therapist/patient/tablet triad, the digital tool was described as a lever for interaction, based on the curiosity that the tablet arouses, particularly in younger individuals, and even in situations of opposition. However, the digital tool was also noted as a potential source of worry for participants when they felt the child mastered it better than they did*: “And besides, I know they can see how clumsy I am with it, that I’m struggling, and they actually like that side of it—like [question from a patient], ‘Oh, you need some help?’ ‘Go on then, show me, smart guy.”* (FG-T6). These shifts in practice and stance could be perceived as either barriers or levers to the app’s usability, depending on the participant.

Additionally, using a digital app required the participants to work differently and adopt new habits. The use of personal identification codes complicated access for some participants. Managing the tablet’s battery life requires planning for recharging to ensure availability during sessions. Participants suggested formalising new behaviours: *“when I arrive in the morning, I* *check everything, if the battery is charged, that way, if there’s a problem, I have time to sort it before the appointment”* (FG-T3). Other suggestions were regular tablet charging, using calendar reminders, and designating a responsible person.

The first barrier mentioned concerned the length of completion with the MFM-Play app, which was longer than the traditional version. However, the consultation duration was also prolonged by the additional monitoring required for the reliability study protocol that the therapists were participating in. The routine use of MFM-Play outside of a research protocol would likely dissipate this barrier: *“I pretty much only use the tablet now, because at first, to practice outside the study, I tried it out on other patients—and then, over time, I quickly got used to it”* (FG-T1). In addition, greater familiarity with the tablet and the app’s functionalities could optimise evaluation duration, which had previously hindered the use of MFM-Play by participants. Support in appropriating the tool could serve as a lever to enhance usability and change the perception of the app as being time-consuming. Lastly, ensuring the work environment has sufficient internet connectivity is essential for the proper usability of MFM-Play during consultations: *“its mainly the internet problem that makes it complicated”* (FG-T7).

#### Perceived lack of usability of the interface.

Participants mentioned difficulties in using the tablet; some did not master basic commands, such as turning off the tablet: “Yeah, once we couldn’t figure out how to turn it off [response from a child] “You have to do it like this—don’t you know how?” “No, I don’t. I don’t have a tablet at home.” (FG-T6). The data export function was unfamiliar to some participants, raising questions about the proper placement of this button in the app interface. Additionally, the three interactive items did not seem fully mastered, as barriers related to their use were resolved by others during discussions, illustrating partial mastery of navigation for these items.

#### Dissatisfaction with the evaluation.

The participants raised several points regarding their satisfaction. The scoring page for each item was considered the least satisfactory visual element of the evaluation. Participants found the colours associated with the ratings from 0 to 3 unintuitive: *“when it comes to rating, 3, which is the best score, is in red [several participants nodded], and it throws me each time”* (FG-T5). Moreover, the fact the patient could see the score made some participants hesitant to share the tool.

To increase the usability of MFM-Play, the participants suggested focusing on patient motivation. They proposed stronger links between evaluation items and the storyline, as well as the option to choose story themes during MFM-Play sessions: *“the stories are centred around the theme of planets, that doesn’t interest everyone, but there was not much choice”* (FG-T2) (e.g., animal world, princesses, soccer, and cartoon characters). In terms of audio, the participants highly appreciated the narrated story version. They found it a real usability lever for MFM-Play: *“and for older ones, the story is not read [nodding from the other participants] and I found they were less engaged in the story”* (FG-T7). Making this feature available across all modules would be welcomed as it would make the evaluation more dynamic and, consequently, less time-consuming. Additionally, they suggested increasing the number of rewards to further align with this motivational goal.

In terms of the most suitable patient profile, the participants thought the MFM-Play was particularly suited to patients aged 6–12 years who have “*a minimal amount of upper limb function*” (FG-T5), defined as the ability to hold the tablet and physically interact with it, such as changing pages. The participants thought the MFM-Play was particularly useful for patients with spinal muscular atrophy, Charcot-Marie-Tooth disease, Duchenne muscular dystrophy, or Friedrich’s ataxia. Naturally, factors such as patient curiosity, compliance, familiarity with digital tools, or preference for the Play version over the traditional version increased the perceived usefulness. Conversely, in cases of cognitive, attention, or motor limitations, the MFM-Play evaluation was more difficult. Participants particularly noted challenges with some children with Duchenne muscular dystrophy starting corticosteroid treatments and those with Steinert’s disease.

## Discussion

The results of this study revealed multifaceted features of MFM-Play adoption, identifying barriers and levers to its integration into clinical practice. Although the app enables practical and efficient completion of the MFM, its implementation requires a shift in rehabilitation professionals’ practices and workflows.

### Ease of use and adaptation to hospital workflows

The main difficulties regarding ease of use related to digital skills and organisational constraints. Adherence to digital technologies and the perception of the app’s ease of use varied among the 14 participants, depending on how comfortable each was with technology. In accordance with the literature, the ease with which the participants administered the evaluation via the app varied [40,41]. This phenomenon may have been exacerbated by the fact that most participants were physiotherapists, a group generally little inclined to use digital technologies in healthcare [40]. One of the predictive factors of an individual’s intention and subsequent behaviour is their attitude towards technology, as conceptualised by the TAM. The interview results highlighted different attitudes among the therapists, leading to varying degrees of acceptance of the MFM Play. Learning to navigate the MFM-Play app, and even the basic functionality of a tablet increased the cognitive load for some therapists during their first uses. The infrequent nature of MFM evaluations could also hinder the integration of the app’s functionality. Some therapists overcame initial difficulties through repetition, driven by the reliability study they were participating in. Repetition at the outset of using a new digital tool is necessary but may be limited by a lack of familiarisation time in demanding clinical settings. Limited time and infrequent training sessions are significant barriers to healthcare professionals’ effective adoption of digital tools [42], thereby limiting therapists’ perceived ease of use according to the TAM. However, some therapists expressed being comfortable with the digital environment, fully integrating the app’s functions, including mastering interactive items and data exporting. These therapists reported greater satisfaction with the app, which should positively influence their future usage behaviour and consequently, according to the TAM, their intention to use the app. During the focus group, these therapists explained features to their colleagues who had not fully understood them. This underscores the value of peer learning or consulting reference evaluators who are comfortable with the app. Therapists who are skilled in digital technology use can be recognised resources for facilitating the integration of new technologies into a therapist team using a socio-constructivist approach [22,40,43–45]. Training programmes enhance therapists’ confidence in navigating digital tools and addressing specific technical issues [25]. To counteract negative conditioning, such as frustration or lack of success using digital technologies, adopting strategies to transform negative experiences into learning and satisfaction opportunities is crucial [42]. To optimise the implementation of MFM-Play, these approaches could be introduced through discussion groups with therapists, targeted training, tutorials, and possibly technical support during the initial setup.

The organisational constraints and technical or infrastructural barriers, such as the need for Wi-Fi installation or retrieving identification codes, highlighted as obstacles to adoption, could be more problematic when used outside research protocols. However, Wi-Fi was available in all ten hospitals, sometimes with relay fittings. These common organisational challenges in the adoption of digital health devices in rehabilitation [22] should not be overlooked, but are likely to diminish as digital tools become more widely deployed in hospitals.

Regarding workflow management, the duration of the MFM assessment is an important issue; it must fit within a consultation length compatible with therapists’ workflows. The MFM-Play took slightly longer to complete than the traditional version; however, it provided benefits in terms of motivation and the assessment experience. Furthermore, this potential barrier to adoption is likely to decrease over time as therapists become more proficient in using the tool. In addition, therapists could adapt the number of stories used during MFM-Play administration to their scheduling constraints, which was not done within the research protocol.

### Perceived utility and satisfaction with the app’s features

To be accepted, a digital tool must also offer utility that meets users’ needs [46]. Utility and usability are distinct concepts, and usability is only one dimension of acceptability. A system may meet all usability criteria but still be considered useless [47,48]. The absence of clear utility for some therapists could be an obstacle to integrating this digital tool. Even the most advanced apps risk non-adoption if they do not offer simplicity and advantages over existing tools [49]. Perceived utility was positive regarding consolidating resources, exporting digital data, and motivating and engaging children, especially those aged 6–12 years. Motivational elements integrated into the app, such as stories, visualising progress through the evaluation, pictures of the items, rewards, and the three interactive items, were valuable assets for maintaining patient engagement. Expanding these features and aligning the stories with patient preferences, as suggested by the therapists, could improve ease of use, utility, and satisfaction. This approach also aligns with the user-centred design of the tool. However, although increasing children’s motivation was acknowledged as a key lever, along with the app’s “icebreaker” role, some therapists did not see the value of using the app to motivate the child. Paediatric assessment specialists might feel their expertise is sufficient to make sessions engaging, as they are accustomed to incorporating play into their therapy. This phenomenon could reduce the incentive to change their practice [27]. Perceived usefulness directly influences the intention to use the technology, which in turn determines actual use [27]. A lack of perceived usefulness of a digital app can lead to reduced adoption, superficial use, or resistance to change [50]. The app may be more readily accepted by therapists who do not have prior experience of the traditional paper MFM.

One of the goals of developing the app was to reduce therapists’ cognitive loads by keeping children focused and engaged throughout the assessment. Although the app seemed to provide a smoother and less energy-intensive session for some therapists, this benefit was not particularly highlighted. This should be re-evaluated outside of a quantitative study to see how the balance between “increased therapist load” and “therapist assistant " evolves when the tool is mastered to a greater extent.

### Changing patient-therapist dynamics

This study highlighted the potential of the MFM-Play app to transform patient-therapist dynamics. Previous work has underscored how integrating structured physical therapy with interactive frameworks can enhance therapeutic engagement and behavioral outcomes [51,52]; our findings similarly reveal how digital interfaces such as MFM-Play can strengthen patient participation and reshape therapist–patient dynamics. By promoting patient engagement and autonomy, the app aligns with modern patient-centred care and patient-partner paradigms [53–57]. It encourages therapists to be more transparent in how they evaluate and score items. This phenomenon was observed in the focus group, where therapists stances differed with regards to their role as facilitators. The therapist/patient/tablet triad may resemble a pedagogical relationship in which the patient can refer to the tablet’s instructions to complete the task. This materialisation of “knowledge” could be a lever or a barrier, depending on the therapist. Integrating such a digital tool requires the therapist to act as a moderator between the app and the patient to optimise the assessment process. Recognising this paradigm shift is essential for measuring user acceptability and identifying the levers needed to optimise the app’s usability. It represents a paradigm shift, making the patient more active in their evaluation and, ultimately, in their care, by understanding how the MFM assessment functions [58]. Thus, when the therapist allows the patient to access the instructions for completion and scoring on the tablet, cognitive processes are no longer the domain of a single individual but are distributed among three entities: the therapist, the patient, and the app, each contributing complementary elements. This aligns with the concept of “*distributed cognition”* [59,60], where cognitive abilities emerge from interactions between people and technological tools, increasing the reliability of the data.

In the long term, the app could be leveraged for remote assessments—an increasingly sought-after solution in the post COVID-19 era [61]. One of the key “promises” of digital health development is to empower patients as co-pilots of their own healthcare journey. Remote monitoring fosters greater patient engagement in their treatment and intermediary consultations reduce the need for hospital visits [62,63].

### Social identity

The introduction of the app altered established therapist practices. Although the MFM scale itself was not modified, and the therapist guided and co-facilitated the assessment, integrating this digital tool disrupted their practices and professional identities more than we anticipated. Some changes were intended by the project, such as increasing patient involvement in the assessment, increasing the transparency of the scale, and reinforcing the standardisation of the assessment completion. For example, some therapists, who believed they correctly administered the MFM assessment, reported being surprised by different patient interpretations of the same item. This led them to reassess their instructions, which may have been overly simplistic regarding the possible motor strategies, while also highlighting the perceived usefulness of this assessment tool. The phenomenon of “drift,” defined as a decrease in the evaluator’s consistency over time [18,19,64,65], may occur in functional assessments through decreasing attention, personal interpretation of the rating system, or forgetting the instructions over time [16,64,65]. Clinical experience is valuable but insufficient to ensure proper reliability [66], as we observed in the MFM-Kinect study [67]. The provision of information via the tablet or the patients themselves could help to reduce drift. However, this can also be a challenge for therapists, leading them to question their skills as evaluators. Furthermore, although the app prevents therapists from using personalised strategies developed during traditional MFM assessments, it could still be “transformed” by users who perceive it as too restrictive, for example by not sharing the screen with the patient. Many apps designed for one purpose can be diverted from their primary function by users [46]. The MFM-Play app seems to require therapists to reinvent their sessions to integrate and “play” with the tool. Digital tools are only accepted once they are perceived as sufficiently beneficial for both the therapist and their practice [68]. Once the technological constraints are overcome, therapists may begin to explore new uses for the tool. The therapists’ experiences with the digital tool—whether through success, challenges, or frustration—will shape their acceptance, use, and integration of the tool into their daily practice.

### Reducing existing barriers

Three recommendations to facilitate the adoption of the MFM-Play app and overcome the identified barriers emerged from this study.

**Provide information, training, and familiarisation time**: Training on using the app and the tablet is essential. One of the determinants of frequent use of a technological tool in paediatric rehabilitation is ease of access to the tool and training, and therapists’ sociodemographic characteristics may play a smaller role [22].

**Support organisational change (logistic and time-related)**: Ensuring infrastructure availability (Wi-Fi, equipment) and integrating reminders (such as for recharging the battery) could overcome logistical barriers.

**Adapt scenarios and increase flexibility**: Adapting certain scenarios and strengthening flexibility around parameters such as evaluation duration would help meet therapists’ needs and improve usability.

### Limitations

This study is contextualised and does not aim to provide explanations with general applicability but seeks to report therapists’ experiences. The mandatory use of the app for participation in the reliability study may have affected the results compared to real-life use. Use in a different context might have yielded different representations and acceptance of the app. Future mixed-methods or longitudinal approaches could be useful to track adoption over time. Also, most participants were physiotherapists. This lack of professional diversity might have influenced the results since other professionals, such as occupational therapists, may have different perspectives on usability and acceptance. The results might have been affected by the “snowball effect,” where more use leads to more usage, and vice versa through feedback loops between perceived acceptance, perceived usefulness, and usage intent [69]. A study conducted in a real-world setting (outside of the research protocol) would be valuable for examining clinical results and therapist and patient satisfaction.

The study focused on the therapists’ perspectives. However, the MFM-Play also changes patient-therapist interactions compared with the traditional paper version. This aspect was addressed in the MFM-Play reliability study through questionnaires. The results will be published separately, along with the reliability results.

## Conclusion

Although the MFM-Play app offers promising opportunities to improve the MFM assessment process, its successful adoption requires establishing clear implementation pathways. This includes structured training for therapists to support their new role within the triadic patient–therapist–tablet relationship, investment in infrastructure, and feedback loops to drive iterative design improvements. Changes in organizational practices that integrate the MFM-play from the outset would push uptake and improve the assessment process.

## Supporting information

S1 Appendix MFM-Play app functionality overview and interview and focus group Guide. (DOCX)
