# Supplementary material for: Barriers and levers to using an app for motor function assessment by therapists in hospitals: A qualitative study of the MFM-Play app
Source: PLoS One. 2026 Jul 31;21(7):e0352856. doi: 10.1371/journal.pone.0352856 (PMC13426978; doi:10.1371/journal.pone.0352856)
Supplement: S1 Appendix — (DOCX) [file pone.0352856.s001.docx]

**Appendix**

**Fig. 1:** Diagram of the sequence of item submissions with the MFM-Play application
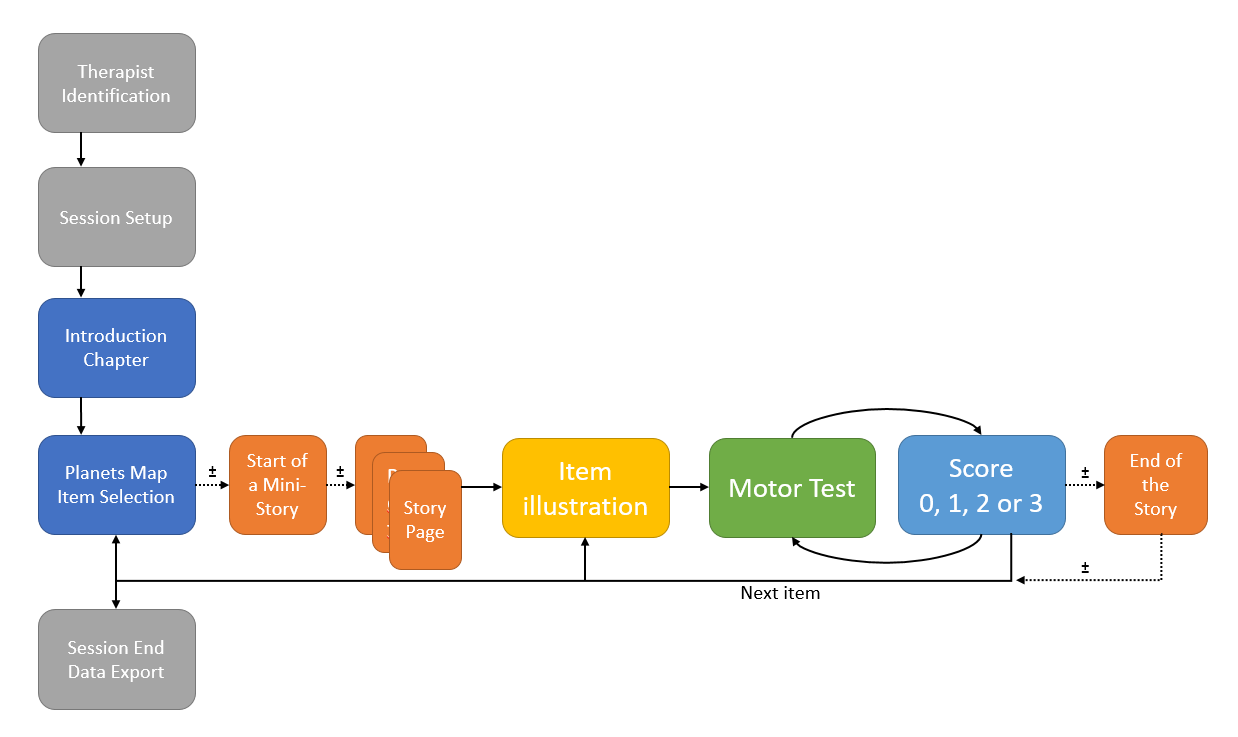


**Appendix, Fig. 2:** Screenshots of the MFM-Play interface


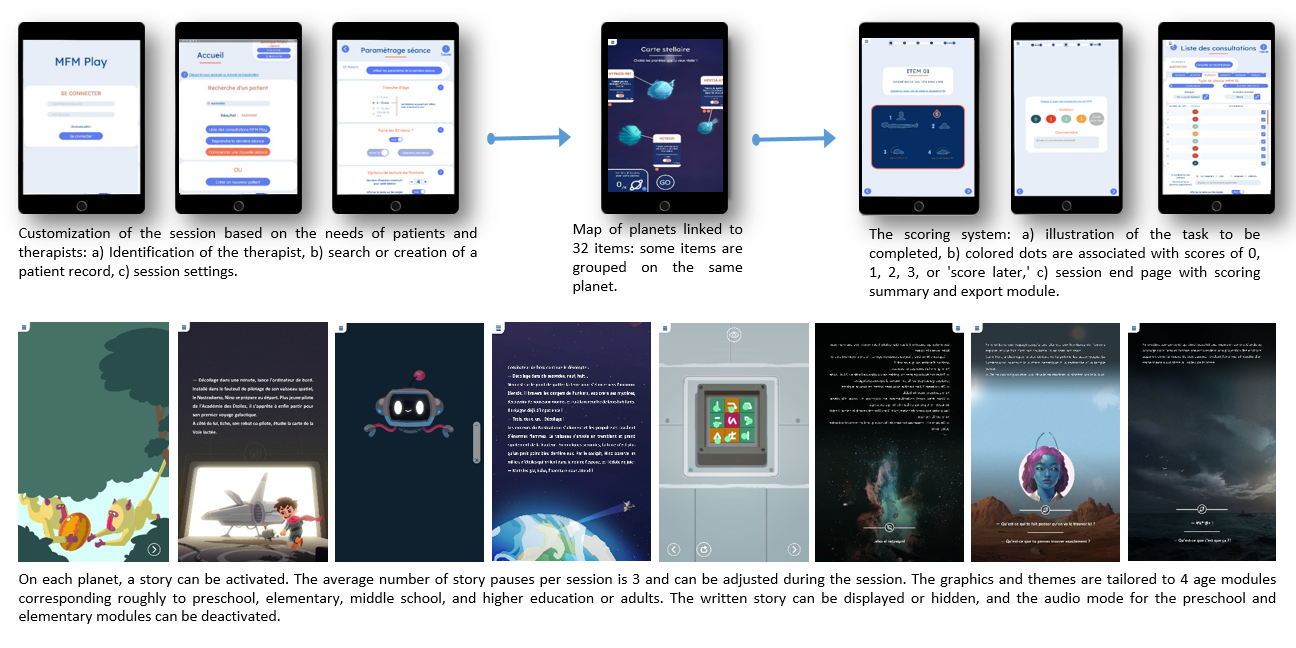


**INTERVIEW GUIDE**

COMMUNICATION CONTRACT:
“Thank you for agreeing to take part in this interview today. I’d like to remind you that I’m a social psychologist on the project, and therefore in charge of this interview. The aim is to get your point of view as an MFM Play user therapist. There are no right or wrong answers, and I won’t pass any judgement, so please feel to share with me any elements you wish, as the aim is to gather your most sincere opinion possible. This should last about 45 minutes but you’re free to stop the interview whenever you like. We can also take breaks if you wish. The interview is anonymous, and the data will only be used for the purposes of this research. This means that your name will not appear at any time, nor will any names of people or towns you may mention to me during this exchange. As I told you last time, and as you can read in the information letter, if possible I would like to record the interview. This will enable me to be an active listener and to transcribe your words sincerely for this research. If you agree, and if you don’t have any questions now, we can start the interview.”

*Start audio recording after communication contract and participants oral agreement*

| THEMES | QUESTIONS | PROMPTS |
| --- | --- | --- |
| Theme 1:  User satisfaction | In general, how satisfied are you with the MFM Play application? | - Conversely dissatisfaction - Ease with digital tools in general - Perceived patient satisfaction |
| Theme 2:  Effectiveness and efficiency | How would you describe the effectiveness of the MFM Play application? | - Effectiveness in the sense of reliability/validity = goal attainment - Efficiency = ease of goal attainment (minimum time and effort) - Perceived effectiveness for the patient (in terms of commitment) |
| Theme 3:  Appropriation of the application | What would you say about how you came to appreciate the MFM Play application? | - Learning conditions - Memorisation conditions - Temporality (habituation): before first use versus over time - Appropriation by the patient? How is it perceived? |
| Theme 4:  Social influence on MFM Play usability | What do other professionals think of the MFM play application? | - Social norms & social influences: opinion perceived by other professionals in the profession? By other members of the department? - How was the change brought about within the department? How was the change in practice perceived? |

**FOCUS GROUP GUIDE**

**COMMUNICATION CONTRACT:**

“Thank you for agreeing to take part in this focus group today. I’d like to remind you that I’m a social psychologist on the project, and therefore in charge of qualitative study. The aim is to get your point of view as an MFM Play user therapist. As you probably know, there was a first phase of individual interviews with therapists who were also MFM Play users. Today, you’re taking part in a slightly different format through this focus group to discuss what your colleagues may have raised as obstacles to using the MFM Play tool. You’re going to share your perceptions of the different themes. Everyone has their own experience and opinion on various themes. Don’t hesitate to express yourself freely, whether you agree or disagree. All I ask is that you show respect listen and be kind to one another. There are no right or wrong answers, and I won’t pass any judgement, so please feel to share together any elements you wish, as the aim is to gather your most sincere opinion possible. I would also ask you to think outside the context of the clinical study, but more in terms of using the tablet in your follow-up consultations.

This should last about 2 hours but you’re free to stop whenever you like. We can also take breaks if you wish. This exchange is anonymous, and the data will only be used for the purposes of this research. This means that your name will not appear at any time, nor will any names of people or towns you may mention to me during this exchange. As I told you last time and as you can read in the information letter, if possible i would like to record the Focus Group. This will enable me to be an active listener and to transcribe your words sincerely for this research. If you agree, and if you don’t have any questions now, we can start the interview.”

*Star audio recording after communication contract and participants oral agreement*

| **THEMES** | **TOOL** | **QUESTIONS** | **PROMPTS** |
| --- | --- | --- | --- |
| **Theme n°1:**  **Practice change coaching** | Presentation of 4 verbatim from different interviews. Presentation of verbatim one after the other  *«it was something i was a bit afraid of: having to make sure the tablet was plugged in, enter the codes and all that, it’s always a bit like that, it always takes a bit of time in the clinic»*  *« I’ll admit that, at first, it took me a while to do it, because I thought, no, but I ... it’s going to be too complicated, it’s going to take too long, I won’t have the time »*  *« I think I got stuck on the beginning, the transition and all that, which wasn’t easy for me »*  *« it’s true that Android is a ... it’s a world where I’ve never ... I’ve never been confronted more than that, probably by choice and also by environment and so I find myself learning a new ... new techniques and it’s a bit ... well it can be a bit disturbing »* | Faced with each of the verbatim presented to you, I will ask you to discuss the elements that helped you in this type of situation?  What factors could have helped you, as a therapist, to deal with the difficulty encountered? | - Integration into non-protocol practice - Projection on someone learning MFM with the tablet - Mastery of digital tools (iOS, android?)/mastery of digital assessment - Self-confidence perceived (attention to context) - A priori - Tablet training |
| **Theme n°2 :**  **Change in patient relationship** | Continuum of participation in evaluation in the form of axes:   - From passive to active patient - From passive to active therapist   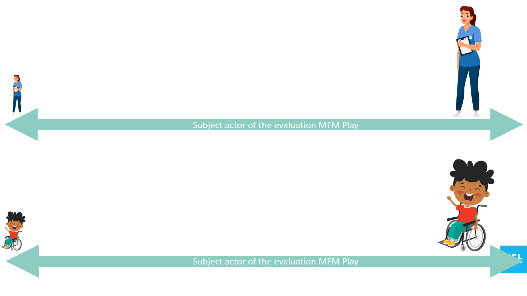 | During the analysis of the interviews, we noticed that the use of MFM Play could question the therapist’s relationship with patient.  I propose an exercise based on these two axes, which represent a continuum of participation from passive to active, one for the patient and the other for the therapist. Using post-it notes, I will ask you to situate yourself on these two axes, explaining how you perceive them in your current practice. | - Comfort with the role - Satisfaction with role - Talking in terms of interaction with the patient - Talk about other situations not mentioned: when the hild wants to take the tablet, what happens? - Sharing the assessment support: what are the consequences (visualization of the rating by the patient)? |
| **Theme n°3:**  **Perceived difficulties for the patient** | Creation of 2 profiles cards  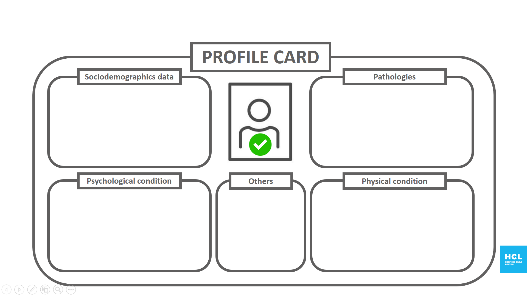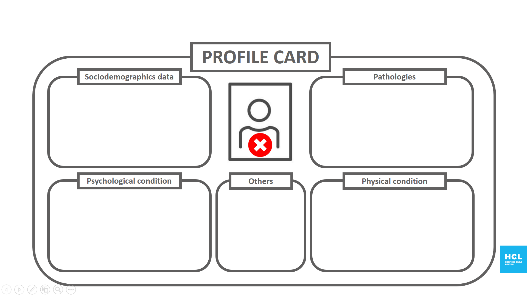 | Using the “profile cards”, I suggest that you fill in the various boxes together, to present the typical patient profile for whom the MFM play is appropriate.  In a second phase, you will be asked to carry out the same exercise with the opposite instructions, the patient for whom the MFM play seems less suitable. | - Sociodemographic data - Pathologies - Psychological condition - Physical condition - Justifying choices |
